# Properly designed femoral stem impactors help to avoid overstuffing and make a second trial reduction unnecessary

**DOI:** 10.1007/s00402-026-06242-2

**Published:** 2026-03-09

**Authors:** Steffen Brodt, Sebastian Rohe, Philipp Knospe, Pablo Sanz-Ruiz, Georgi Wassilew, Georg Matziolis

**Affiliations:** 1 Orthopaedic Department, Jena University Hospital, Campus Eisenberg Klosterlausnitzer Straße 81, 07607 Eisenberg, Germany; 2 Department of Orthopaedic Surgery, General University Hospital Gregorio Marañón, Madrid, Spain; 3 Center for Orthopaedics, Trauma Surgery and Rehabilitation Medicine, University Medicine Greifswald, Greifswald, Germany

**Keywords:** THA, Total hip arthroplasty, Hip stem, Subsidence, Overstuffing, Navigation

## Abstract

**Introduction:**

For cementless implantation of hip stems, it is very important that the original stem fits exactly into the femoral prosthesis bed previously shaped using a stem rasp. If the geometry of the stem rasp does not match the prosthesis geometry, this can result in either overstuffing or post-sintering of the original stem in relation to trial reduction with the stem rasp. Overstuffing results in leg lengthening, while subsidence of the stem results in shortening with the risk of dislocation and impingement. Trial reduction with the original stem and trial head can prevent this, but is associated with additional soft tissue trauma and a longer operating time.

**Materials and methods:**

Three groups were prospectively randomized. Group 0 was treated with the Fitmore B stem and the conventional rasp system, Group 1 with the Optimys stem and Group 2 with the Fitmore B stem with a new, optimized rasp system via a minimally invasive posterolateral approach. Intraoperatively, the differences in leg length and offset between trial reduction with the stem rasp and the original stem were recorded using a hip navigation system.

**Results:**

The conventional rasp system led to significant overstuffing (1.2 ± 0.5 mm, *p* = 0.024) with the Fitmore B stem, compared with the instruments of the Optimys stem. In contrast, the optimized rasp system with the Fitmore B stem resulted in an equally precise fit of the endoprosthesis to that achieved with Optimys.

**Conclusion:**

There are relevant differences in the fit of rasp and original stem between different manufacturers and system evolutions. In order to ensure reproducible comparability of the fit of the original stem to the trial rasp, it is imperative that trial rasps are optimally adapted to the stems. This is an important way to avoid trial reduction after implantation of the original stem.

**Trial registration:**

This study was registered in the German Clinical Trials Register (Deutsches Register Klinischer Studien) with the registration number DRKS00026749.

## Introduction

In order to test the parameters of leg length, impingement-free range of motion, dislocation stability and soft tissue tension (through telescoping), a trial reduction is usually carried out when performing total hip arthroplasty (THA). This is done with the appropriate stem rasp and the selected trial head. With modern implant designs, several offset versions of the trial neck and different head lengths are available, usually short (S), medium (M), long (L), and extra-long (XL). If the parameters checked intraoperatively are correct and the surgeon is satisfied with the visual and haptic results obtained, the trial components are removed and the original prosthesis is implanted. The most common implantation method is the cementless press-fit technique. 97% of stems are now implanted cement-free using this technique in the USA, 77% in Germany, 61% in Australia, and even 33% in countries such as Sweden that traditionally prefer the use of cement [1]. The bony bed into which the original prosthesis is implanted must first be pre-shaped using rasps of different sizes. There are various manual techniques for this [2–4]. Robot-assisted techniques gained prominence due to their precisely fabricated prosthesis bed, but were abandoned again due to soft tissue-associated complications [5–7]. For the reliable reproducibility of the parameters tested in the trial reduction position, the identical fit of the original stem compared to the trial prosthesis is absolutely essential [8, 9]. If the stem and trial do not match exactly, so-called overstuffing or subsidence of the prosthesis into the femoral medullary cavity may occur [10]. As a result, the parameters determined in the first trial would no longer be correct. This would result in either a lengthening of the operated leg or laxity of the joint, which would necessitate a new trial reduction, e.g., with a different or adapted head length [11–16]. However, as every trial reduction (and dislocation) inevitably leads to damage to the soft tissues, including the muscles, and prolongs the duration of the operation, these should be kept to a minimum.

The manufacturers of endoprostheses are aware of this problem and are doing their best to match the geometry of the rasp systems to the original stems to avoid a stem sitting proud or too deep [17]. To this end, adaptations also are being made to rasp systems already on the market to enable an optimized and more precise fit.

The aim of the present study is to investigate the accuracy of fit of different short-stem systems in relation to the respective stem rasps. Given an exact fit from original to trial, a muscle-traumatizing and time-consuming second trial reduction can be dispensed with.

## Methods

**Fig. 1:**
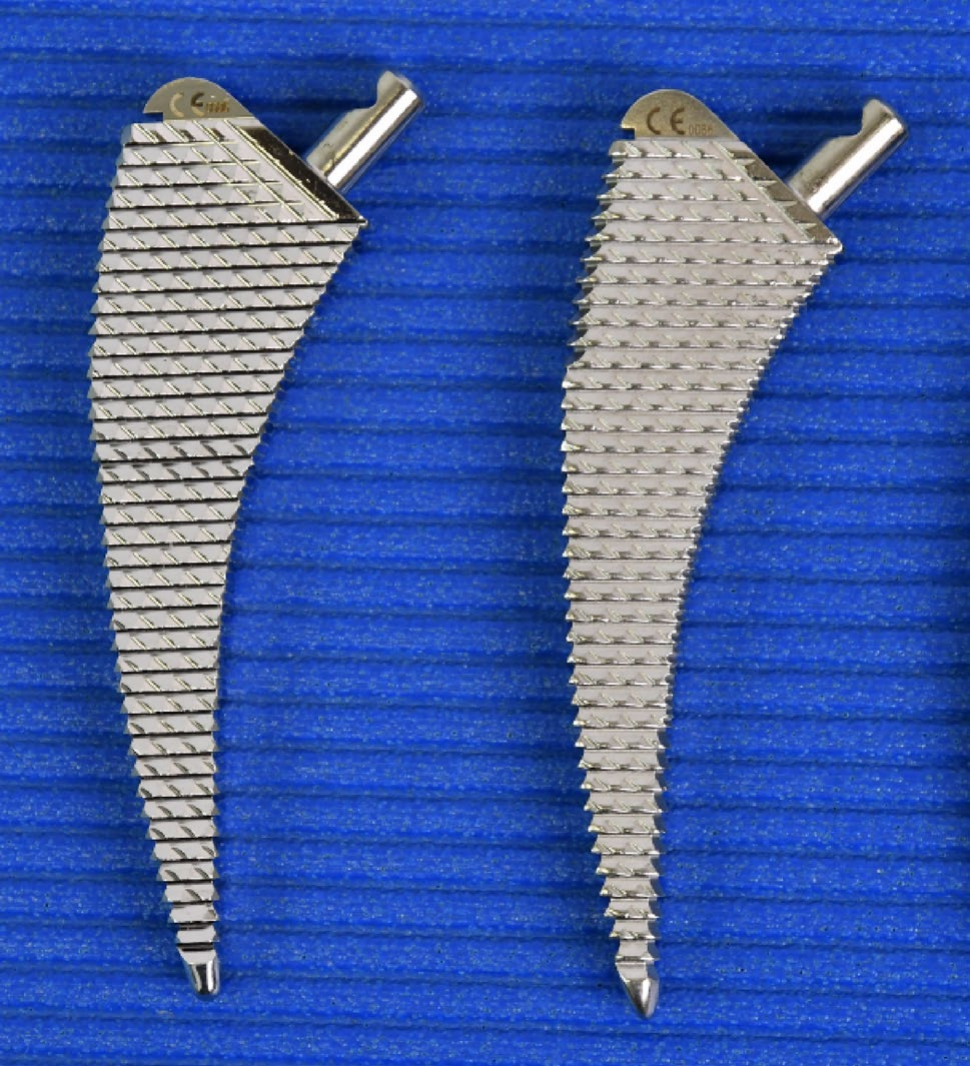
Left: new Fitmore rasp; right: conventional fitmore rasp

This is a prospective randomized study. The Fitmore stem size B (Warsaw, IN, USA) was used in combination with two different stem rasp designs. The “old” design of the rasp was compared with a “new” one, that is thicker at the proximal end to mimic the proximal coating of the original stem (Fig. 1).

The study was registered in the German Clinical Trials Register (Deutsches Register Klinischer Studien) with the registration number DRKS00026749. The responsible ethics committee gave its approval on April 29, 2021, with the number 2021-2165-BO. A power analysis (g*power, one-way ANOVA, multiple groups, Heinrich Heine University) was conducted before the start of the study. This was based on the assumption that the minimum expected difference between rasp and implant fit was 3 mm, with a standard deviation of 2 mm. The significance level was set at 0.05 and the power at 0.8.

Patients with primary coxarthrosis who were scheduled to be fitted with a short stem and who had consented to the study after prior informed consent were included. Patients with secondary coxarthrosis, e.g. following trauma or femoral head necrosis, were excluded, as were patients with osseous anomalies, tumors and osteoporosis. Pregnant women were excluded, as were patients < 18 years of age. All patients were operated via a minimized posterolateral approach.

**Fig. 2:**
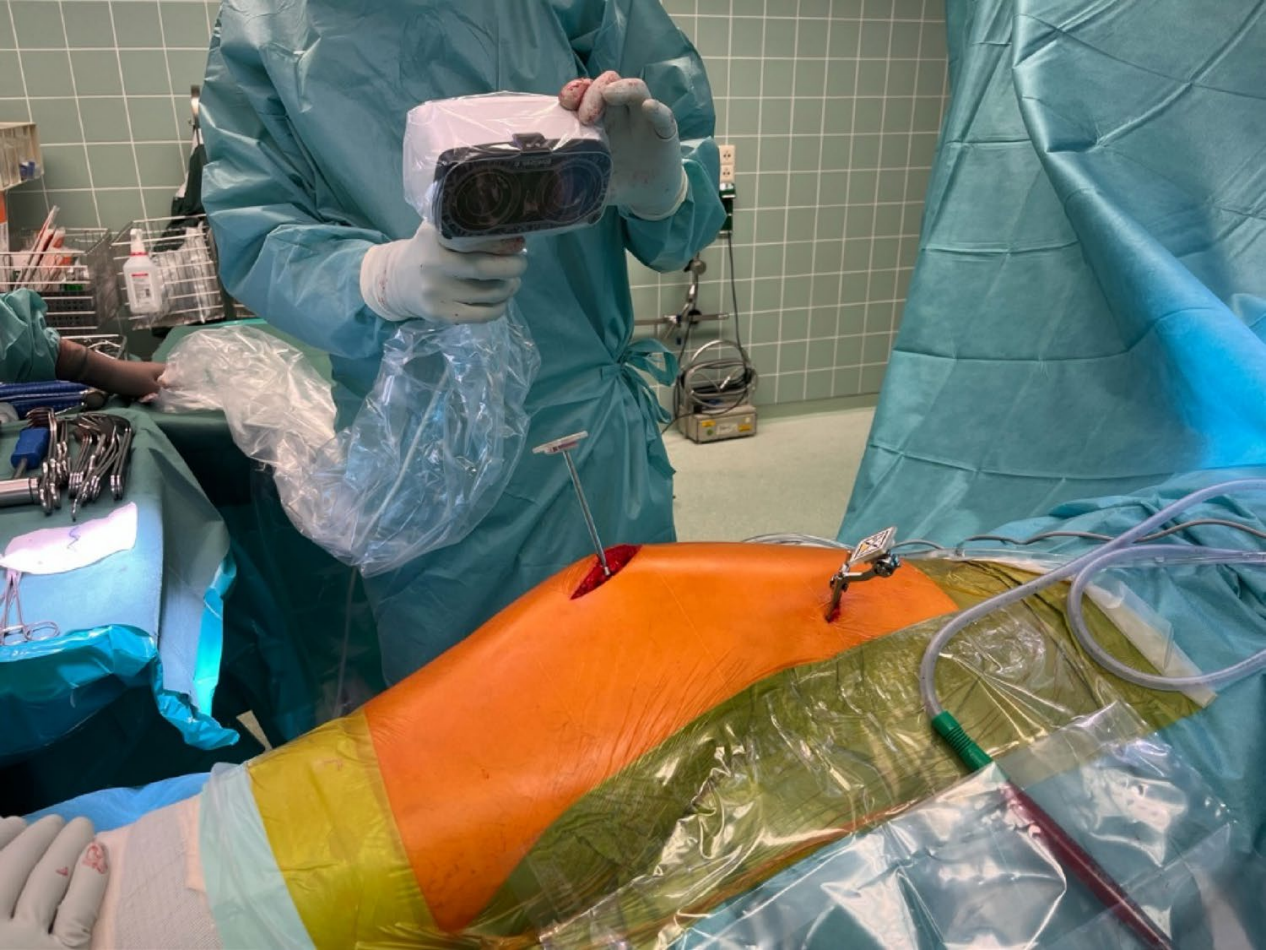
Intraoperative navigation using a postero-lateral approach

The intraoperative data were collected using the NaviSwiss (Brugg, Switzerland) hip navigation system. This is a camera-supported, imageless navigation system that has been on the market since 2018 [18, 19]. When the hip arthroplasty was implanted, the offset and leg length were measured and recorded using the navigation system NaviSwiss at the time of reduction with the trial rasp (Fig. 2). After implantation of the original prosthesis and renewed reduction, the same measurement was carried out and recorded again. The difference between the two values was evaluated as the accuracy of the prosthesis fit compared to the trial rasp. The inclination and anteversion of the cup were also recorded by the navigation device.

**Fig. 3:**
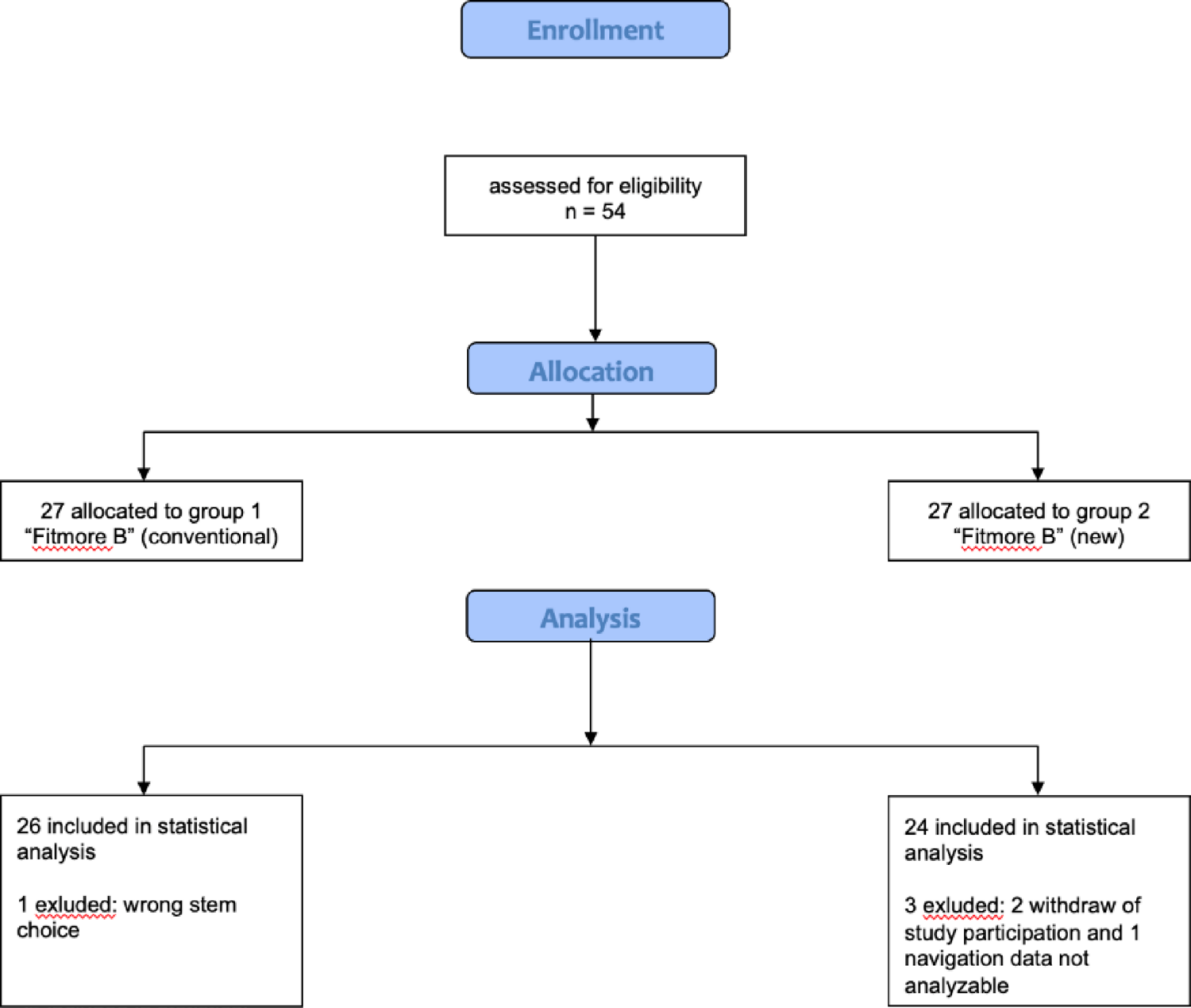
Flow chart

The patients were allocated to 2 randomized groups. Group “1” was assigned the “Fitmore B” stem from Zimmer (Warsaw, IN, USA) with the old, previous stem rasp design and Group “2” was assigned the Fitmore B stem (Zimmer) with the new, revised stem rasp design. Evaluable intraoperative navigation data were available from 50 patients. In Group 1 data from 26 patients could be analyzed, in Group 2 from 24 patients (flow chart, Fig. 3).

A total of 23 women and 27 men were included in the study and operated on by 2 experienced senior surgeons (GM and SB). The right hip was operated on 30 times, the left 20 times. The average age of the patients was 67.8 ± 8.3 years. The youngest patient was 45, the oldest 84 years old. The average operation time was 55.9 ± 12.8 min (35–90 min). The patients were on average 171.0 ± 8.3 cm (150–188 cm) tall and weighed 80.8 ± 13.1 kg (62–120 kg). The average BMI was 27.8 ± 4.9 kg/m^2^ (22.3–47.6 kg/m^2^).

## Results

Average cup inclination measured intraoperatively by the navigation system was 41.6 ± 4.2° (32–52°), while anteversion was 21.5 ± 4.4° (11–34°). The median implanted cup size was 54 mm (48–62 mm).

In Group 1 (Fitmore “old” rasp screen), the median implanted stem size was 5 (2–7) and in Group 2 (Fitmore “new” rasp screen) it was 6 (1–11). No significant differences were found in any of the aforementioned parameters.

The difference between the trial and the original stem (= inaccuracy of the stem rasp) was 1.6 ± 1.9 mm (−3–6 mm) in Group 1 (Fitmore “old”) and 0.7 ± 2.0 mm (−3–4 mm) in Group 2 (Fitmore “new”). This difference did not reach statistical significance (*p* = 0.13).

**Fig. 4:**
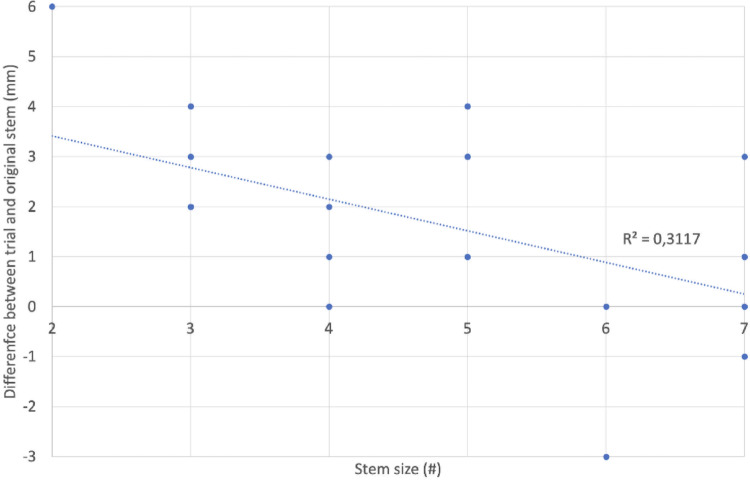
Difference between trial and original stem (mm) depending on stem size

But in group 1, there was a significant linear correlation between the rasp size and the inaccuracy of the rasp (*R* = −0.558, *p* = 0.003). The smaller the rasp, the greater the deviation of leg length from the original stem (approx. 0.6 mm more deviation per smaller size, Fig. 4). At a size of 6 and above, there was no longer any difference between the rasp and the original stem.

In contrast to leg length, no group difference could be shown for the offset (*p* = 1).

## Discussion

The main result of the present study is that the fit of the original stem is less accurate in Group 1 (Fitmore “old” rasp screen) than in Group 2 (Fitmore “new” rasp screen) without reaching statistical significance.

The identical fit of the implanted original stem in comparison to the trial rasp, previously checked during the surgical procedure by means of trial reduction, is a key quality feature of the instruments. When implanting total hip arthroplasty, the surgeon must rely on this to ensure the desired geometry in terms of leg length, dislocation stability and impingement-free range of motion. Overstuffing leads to an undesired increase in leg length, while subsidence of the original stem results in the need to select a longer head or even a larger stem. This inevitably leads to renewed muscle trauma, which should be avoided, especially in the context of minimally invasive approaches.

In order to guarantee this precision, the manufacturer needs to match the rasp design exactly to the original prosthesis.

**Fig. 5:**
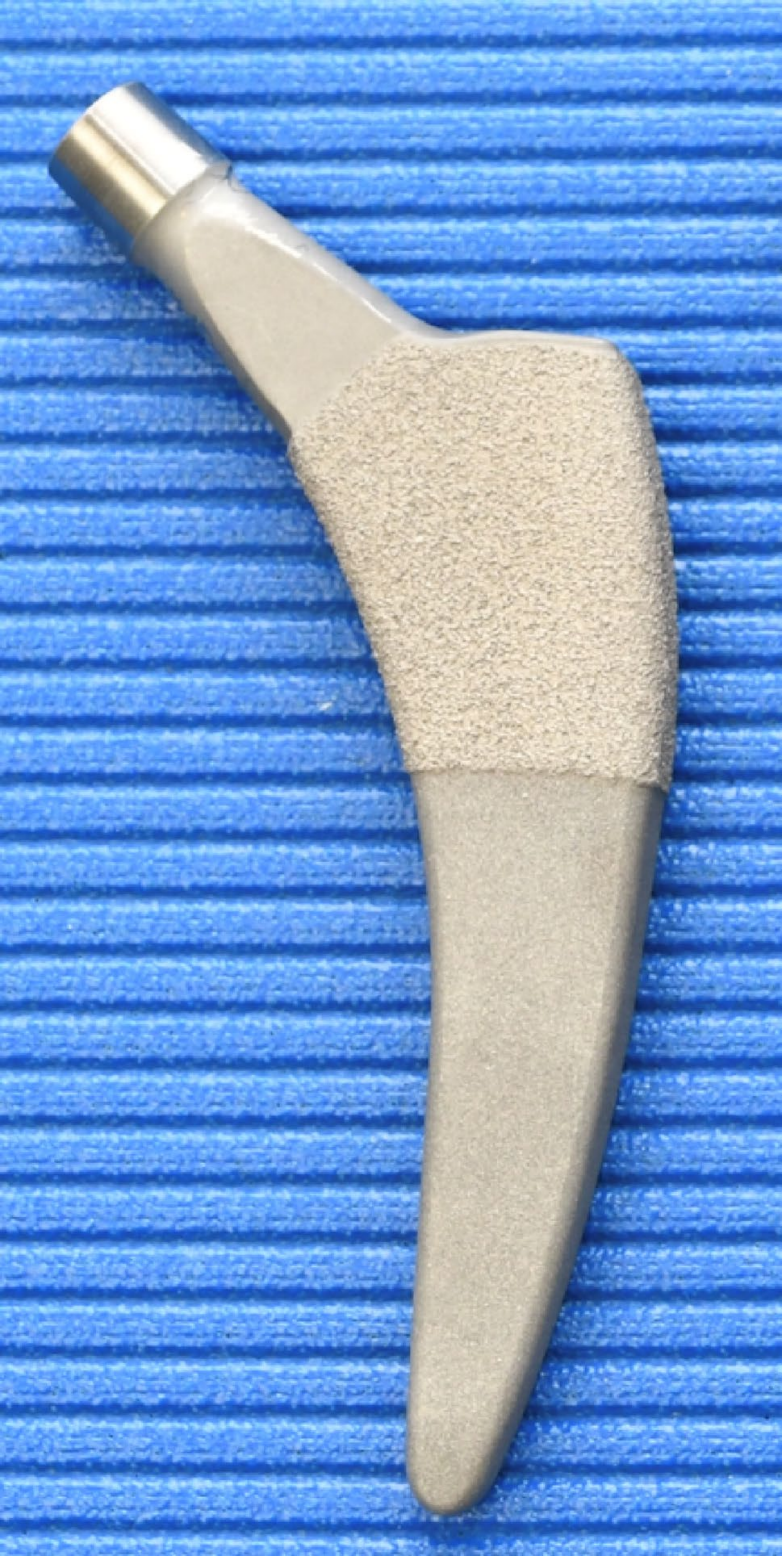
Fitmore stem (Zimmer, Warsaw, IN, USA)

The Fitmore stem from Zimmer has a widening with a macrostructure in the proximal area, which is designed to ensure good anchorage and incorporation into the bony bed (Fig. 5). This change in caliber is not reflected in the original (“old”) rasp design. Therefore, in the authors’ clinical experience, overstuffing often occurred. In other words, the original stem did not penetrate as deeply because the prosthesis bed was not adequately rasped out and the cementless original stem clamped too early. This rasp system is depicted by Group 1 in this study. Meanwhile, rasps are available for the Fitmore stem that adequately reproduce the wider proximal design (Fig. 1). These are available from size 1 to size 5. From size 6 upwards, conventional stem rasps must be used. This study shows that overstuffing mainly occurs in the small rasp sizes of the conventional Zimmer Fitmore B rasp system. This undesirable effect disappears with larger prostheses. With the new rasp system of the Fitmore B stem revised up to size 5, a very reliable fit of the original stem in the previously prepared femoral bed can be assumed. It is possible that the femoral bed prepared by the new Fitmore rasps up to size 5 was sufficiently wide to adequately reproduce the proximal widening of the large prostheses. The old rasp system can therefore be fallen back on from size 6 upwards.

One limitation of the present study is the measurement principle of rasp accuracy using a navigation system. Here, an absolute accuracy of the individual measurement of approx. 2 mm can be assumed [19]. However, this is not relevant for our study, as the measurement was performed with the same cup and leg position. The error in determining the difference between the rasp and implant will therefore be significantly less than 1 mm. Another limitation is the difficulty in standardizing the stem preparation. The preparation was carried out according to the manufacturer’s instructions; the force applied and the number of hammer blows until the preparation was completed were not measured.

The difference between stem preparation and the final position of the implant is of practical clinical relevance and might be determined by manufacturers as part of approval studies in future.

## References

[1] Endoprothesenregister Deutschland (EPRD) Jahresbericht 2022, zu beziehen über die Website des Endoprothesenregisters Deutschland https://www.eprd.de/de/, Zugriff am 27.09.2023 n.d

[2] Hjorth MH, Stilling M, Søballe K, Nielsen PT, Christensen PH, Kold S (2016) Preparation of the femoral bone cavity in cementless stems: broaching versus compaction. Acta Orthop 87:575–582. 10.1080/17453674.2016.1244958 27759486 10.1080/17453674.2016.1244958 PMC5119439

[3] Okowinski M, Hjorth MH, Mosegaard SB, Jürgens-Lahnstein JH, Storgaard Jakobsen S, Hedevang Christensen P et al (2021) Ten-year comparison of two different techniques for femoral bone cavity preparation-broaching versus compaction in patients with cementless total hip arthroplasty : a randomized radiostereometric study of 30 total hip arthroplasties in 15 patients operated bilaterally. Bone Jt Open 2:1035–42. 10.1302/2633-1462.212.BJO-2021-0152.R1 34865512 10.1302/2633-1462.212.BJO-2021-0152.R1 PMC8711659

[4] Husseini A, Nooh A, Tanzer D, Smith K, Tanzer M (2018) Washing the femoral canal results in more predictable seating of a short, tapered femoral stem. J Arthroplast 33:3220–3225. 10.1016/j.arth.2018.05.047 10.1016/j.arth.2018.05.047 30041990

[5] Honl M, Dierk O, Gauck C, Carrero V, Lampe F, Dries S et al (2003) Comparison of robotic-assisted and manual implantation of a primary total hip replacement. A prospective study. J Bone Joint Surg Am 85:1470–8. 10.2106/00004623-200308000-00007 12925626 10.2106/00004623-200308000-00007

[6] Lim S-J, Kim S-M, Lim B-H, Moon Y-W, Park Y-S (2013) Comparison of manual rasping and robotic milling for short metaphyseal-fitting stem implantation in total hip arthroplasty: a cadaveric study. Comput Aided Surg 18:33–40. 10.3109/10929088.2012.744430 23253159 10.3109/10929088.2012.744430

[7] Nishihara S, Sugano N, Nishii T, Miki H, Nakamura N, Yoshikawa H (2006) Comparison between hand rasping and robotic milling for stem implantation in cementless total hip arthroplasty. J Arthroplasty 21:957–966. 10.1016/j.arth.2006.01.001 17027537 10.1016/j.arth.2006.01.001

[8] Barink M, Meijers H, Spruit M, Fankhauser C, Verdonschot N (2004) How close does an uncemented hip stem match the final rasp position? Acta Orthop Belg 70:534–539 15669452

[9] Varini E, Cristofolini L, Traina F, Viceconti M, Toni A (2008) Can the rasp be used to predict intra-operatively the primary stability that can be achieved by press-fitting the stem in cementless hip arthroplasty? Clin Biomech 23:408–414. 10.1016/j.clinbiomech.2007.11.002 10.1016/j.clinbiomech.2007.11.002 18068878

[10] Hofstaedter T, Najfeld M, Fessel G, Orlandini LC, Hube R (2020) Discrepancy of trial rasp and femoral stem relative position within the femoral canal of a coated tapered system: an intraoperative, intrapatient controlled study. Arthroplast Today 6:819–824. 10.1016/j.artd.2020.07.032 33015261 10.1016/j.artd.2020.07.032 PMC7522528

[11] Bahl JS, Arnold JB, Saxby DJ, Taylor M, Solomon LB, Thewlis D (2023) The effect of surgical change to hip geometry on hip biomechanics after primary total hip arthroplasty. J Orthop Res 41:1240–1247. 10.1002/jor.25455 36200414 10.1002/jor.25455 PMC10947254

[12] García-Rey E, García-Cimbrelo E (2016) Abductor biomechanics clinically impact the total hip arthroplasty dislocation rate: a prospective long-term study. J Arthroplasty 31:484–490. 10.1016/j.arth.2015.09.039 26489381 10.1016/j.arth.2015.09.039

[13] Kayani B, Pietrzak J, Hossain FS, Konan S, Haddad FS (2017) Prevention of limb length discrepancy in total hip arthroplasty. Br J Hosp Med 78:385–390. 10.12968/hmed.2017.78.7.385 10.12968/hmed.2017.78.7.385 28692359

[14] Flecher X, Ollivier M, Argenson JN (2016) Lower limb length and offset in total hip arthroplasty. Orthop Traumatol Surg Res 102:S9-20. 10.1016/j.otsr.2015.11.001 26797005 10.1016/j.otsr.2015.11.001

[15] Gheewala RA, Young JR, Villacres Mori B, Lakra A, DiCaprio MR (2023) Perioperative management of leg-length discrepancy in total hip arthroplasty: a review. Arch Orthop Trauma Surg 143:5417–5423. 10.1007/s00402-022-04759-w 36629905 10.1007/s00402-022-04759-w

[16] Mavčič B, Antolič V (2021) Cementless femoral stem fixation and leg-length discrepancy after total hip arthroplasty in different proximal femoral morphological types. Int Orthop 45:891–896. 10.1007/s00264-020-04671-1 32572540 10.1007/s00264-020-04671-1

[17] Kim C-H, Lee SJ, Aditya K, Kim HY, Yoon KS, Yoon PW (2019) Incidence of a stem sitting proud of a proximally coated cementless tapered wedge stem. J Orthop Translat 19:118–125. 10.1016/j.jot.2019.02.002 31844619 10.1016/j.jot.2019.02.002 PMC6896482

[18] Hasegawa M, Naito Y, Tone S, Sudo A (2022) Accuracy of a novel accelerometer-based navigation (Naviswiss) for total hip arthroplasty in the supine position. BMC Musculoskelet Disord 23:537. 10.1186/s12891-022-05495-3 35658945 10.1186/s12891-022-05495-3 PMC9166425

[19] Scholes C, Schwagli T, Ireland J (2023) CT validation of intraoperative imageless navigation (Naviswiss) for component positioning accuracy in primary total hip arthroplasty in supine patient position: a prospective observational cohort study in a single-surgeon practice. Arthroplasty 5:63. 10.1186/s42836-023-00217-z 38049889 10.1186/s42836-023-00217-z PMC10696686

